# Age-related effects on the modulation of gut microbiota by pectins and their derivatives: an *in vitro* study

**DOI:** 10.3389/fmicb.2023.1207837

**Published:** 2023-07-05

**Authors:** Fangjie Gu, Nadja Larsen, Nélida Pascale, Sune Allan Petersen, Bekzod Khakimov, Frederique Respondek, Lene Jespersen

**Affiliations:** ^1^Department of Food Science, Faculty of Science, University of Copenhagen, Frederiksberg, Denmark; ^2^CP Kelco ApS, Lille Skensved, Denmark; ^3^CP Kelco, Levallois-Perret, France

**Keywords:** prebiotic, pectin, inulin, elderly, aging, gut microbiota, short chain fatty acids

## Abstract

**Introduction:**

The present study investigates whether supplementation with pectin-type polysaccharides has potential to improve aging-associated dysbiosis of the gut microbiota. The influence of different types of pectins on the gut microbiota composition and short-chain fatty acids (SCFAs) profiles of elderly was compared to younger adults.

**Methods:**

Pectins studied included a pectin polysaccharide (PEC), a partially hydrolyzed pectin (PPH), and a pectin oligosaccharide (POS). Additionally, inulin was used as a reference prebiotic substrate. Individual fecal samples were collected from healthy elderly volunteers (70–75 years) and younger adults (30–35 years). *In vitro* fermentations were performed using the CoMiniGut model with controlled temperature and pH. Samples were withdrawn at baseline and after 24 h fermentation for measurement of SCFAs production and microbiota composition by 16S rRNA gene sequencing.

**Results and Discussion:**

The results showed that fermentations with PEC and PPH resulted in a specific stimulation of *Faecalibacterium prausnitzii* regardless of the age groups*. Collinsella aerofaciens* became a dominating species in the young adult group with fermentations of all three pectins, which was not observed in the elderly group. No significant differences in SCFAs production were found among the pectins, indicating a high level of functional redundancy. Pectins boosted various bacterial groups differently from the reference prebiotic substrate (inulin). We also found inulin had reduced butyrogenic and bifidogenic effects in the elderly group compared to the younger adult group. In conclusion, the *in vitro* modulating effects of pectins on elderly gut microbiota showed potential of using pectins to improve age-related dysbiosis.

## 1. Introduction

The large microbial community residing in the human intestine, often named as human gut microbiota, plays a vital role in maintaining the health status of human beings (Holscher, 2017). The gut microbiota is dynamic over lifetime, with an assembly phase starting just after birth for about 2 years, followed by relative stability during adulthood until distinct changes appearing with aging (Castanys-Muñoz et al., 2016; Greenhalgh et al., 2016). Despite interindividual variations, several aging-associated bacterial groups have been identified (An et al., 2018). Major shifts included a lower abundance of *Bifidobacterium* and a higher abundance of *Enterobacteriaceae* family being found in elderly fecal microbiota compared to young adults (An et al., 2018). A functional metagenomic analysis reported aging-related loss of genes responsible for saccharolytic potential and short-chain fatty acids (SCFAs) production, accompanied by an increased proteolytic activity (Rampelli et al., 2013). Although the exact mechanism behind the alterations in the elderly gut microbiota has not been fully unraveled, changes in diet and physical activity, antibiotic administration and altered gut physiology are supposed to be contributors (Buford, 2017). Taking into consideration that the elderly population is rapidly increasing (projected to reach 1.4 billion globally by 2030) as well as a generally longer lifespan of individuals (WHO, 2021), a healthy gut microbiota in elderly is of increasing significance. Dietary supplementation with prebiotics could be a promising approach to beneficially modulate the gut microbiota and thereby to improve health conditions of the elderly. According to the expert consensus of the International Scientific Association for Probiotics and Prebiotics (ISAPP; Gibson et al., 2017), a prebiotic is defined as “a substrate that is selectively utilized by host microorganisms conferring a health benefit.” Most well-known prebiotics so far include β-fructans (fructo-oligosaccharides (FOS) and inulin), and other oligosaccharides from various origins, e.g., galacto-oligosaccharides (GOS), arabino-oligosaccharides and more recently human milk oligosaccharides (HMOs; Roberfroid et al., 2010; Gibson et al., 2017). The benefits of inulin on improvement of bowel function is the first authorized health claim for prebiotics in Europe following a positive European Food Safety Authority (EFSA) opinion (EFSA Panel on Dietetic Products and Allergies, 2015). Inulins are polysaccharides composed of linear β(2 → 1)-linked fructose with a terminal α-glucose (EFSA Panel on Dietetic Products and Allergies, 2015). Fermentation of inulin in the gut promotes the growth of beneficial microbes, especially *Bifidobacterium* spp., inhibits enteropathogenic microorganisms and generates abundant SCFAs, especially butyrate (González-Herrera et al., 2015; Teferra, 2021). *Bifidobacterium* is the most commonly recognized genus with myriad benefits, e.g., healthy gut microbiota modulation, immunomodulation, anti-cholesterolemic effects, and protection against pathogens (Roberts et al., 2020). Butyrate is one key SCFAs in regulating the immune system and maintaining a healthy epithelial barrier (Baxter et al., 2019).

Recently, pectins have been suggested as a type of emerging prebiotic due to increasing scientific evidence on their capacities to selectively modulate human gut microbiota (Pascale et al., 2022; Rastall et al., 2022). Pectins are complex heteropolysaccharides that are naturally present in plant cell walls. Traditionally, pectins are extracted from fruit and vegetable sources, e.g., citrus, apple, and sugar beet, to be applied in the food industry as gelling or thickening agents (Elshahed et al., 2021). Pectins are considered as dietary fibers, as they pass indigestible through the upper gastrointestinal tract (Ferreira-Lazarte et al., 2019), and are fermented by the human gut microbiota resulting in the production of SCFAs (Holloway et al., 1983; Holscher, 2017). Molecular structures of pectins are varied, depending on their origins and extraction methods (Naqash et al., 2017; Pascale et al., 2022). Although not fully elucidated, a direct link between the structures of non-digestible carbohydrates and their functional effect on the gut microbiota has been reported (Rastall et al., 2022). In a recent study, the impact of pectin structures on the composition of the human gut microbiota was investigated using an *in vitro* experimental setup (Larsen et al., 2019). Promotion of *Faecalibacterium prausnitzii*, *Bifidobacterium* spp., and several other health-related bacterial groups were found to be associated with the structural features of pectins, including sugar composition and degree of esterification (Larsen et al., 2019). Fermentation of the high-methylated citrus pectin stimulated higher abundances of *F. prausnitzii* and resulted in higher production of propionate (Larsen et al., 2019). Furthermore, differences in molecular weight (MW) of pectins led to different fermentation effects, although MW was not covered by Larsen et al. (2019). For instance, *Bifidobacterium* spp., equipped with more exo-acting enzymes, are known to preferentially utilize oligosaccharides of lower MW (Sarbini and Rastall, 2011; Rastall et al., 2022). Likewise, an increased bifidogenic effect of lower MW pectins in comparison with pectin polysaccharides was observed in several *in vitro* studies and at least one human study (Olano-Martin et al., 2002; Gómez et al., 2016; Ferreira-Lazarte et al., 2018).

Limited number of studies have explored the ability of pectins to modulate the elderly gut microbiota. Wilkowska et al. (2021) studied the prebiotic effect of a pectin oligosaccharide (POS) derived from apple in relation to the different ages of the fecal donors, and concluded that the donors´ age was a determinant on the growth of lactic acid bacteria, SCFAs production, and lactic acid profiles from POS fermentation. Another *in vitro* fermentation study compared the effects of POS from lemon peel and fructo-oligosaccharide (FOS) on pooled fecal inoculum of elderly, and found similar microbial profiles and butyrate production (Míguez et al., 2020). However, the aforementioned study lacks a reference assessment with a fecal inoculum from younger adults under a similar fermentation setting. A recent human intervention study with elderly and younger adults found that a daily supplementation of 15 g of sugar beet pectin for 4 weeks resulted in no significant changes in the fecal microbiota profiles or metabolic activity for neither of the groups (An et al., 2019).

Previous studies have explored either the impact of pectins with different structural characteristics or age of fecal donors on the gut microbiota composition and SCFA production (Larsen et al., 2019; Wilkowska et al., 2021). However, there is still a need to investigate how molecular sizes of pectins influence the human gut microbiota and, especially, how they differentially impact age-related differences in microbiota composition, particularly linked to the microbiota of the elderly. The aim of the current study was therefore to investigate how molecular sizes of HM citrus-derived pectins, i.e., a pectin polysaccharide (PEC), a partially hydrolyzed pectin (PPH), and a pectin oligosaccharide (POS), modulate the gut microbiota of elderly vs. younger adults, using an *in vitro* colon model. The main effects driven by the fermentation of the pectins in terms of relative abundances and diversity of the gut microbial community, as well as the cumulative production of SCFAs were evaluated and compared with chicory inulin (INU) as a reference prebiotic substrate.

## 2. Materials and methods

### 2.1. Substrates

Lemon pectin (PEC) with a degree of methyl-esterification of 68.2%, partially hydrolyzed pectin, also called modified pectin (PPH), and pectic oligosaccharides (POS) were provided by CP Kelco ApS (Denmark). Both PPH and POS were derived from the same batch of PEC, but they were produced to have clear differences in MW. PEC had the largest average MW of 419 kDa (76.7% mass recovery), followed by PPH with an average MW of 24 kDa (78.6% mass recovery). POS had the smallest molecules, with an average MW of 2.3 kDa of the major fraction (55.1% mass recovery). Additionally, POS also contained a smaller fraction with an average MW of 0.4 kDa (24.1% mass recovery) and a minor fraction of large molecules with average MW of 372 kDa (13.5% mass recovery). More details of the absolute MW and corresponding weight fraction of the three pectins were shown in Supplementary Figure 1. Commercial inulin (INU) from chicory (purity > 95%) was purchased from Carbosynth Limited (United Kingdom) and included in the experimental setup as a reference.

### 2.2. Determination of molecular weight by size exclusion chromatography

The molecular weight (MW) distribution of PEC, PPH and POS were analyzed by injecting 100 μL of 2.5 mg/mL substrate to Agilent 1,260 Infinity II liquid chromatography system (Agilent, United States) equipped with PSS precolumn 10 μm 8 × 50 mm, PSS Suprema Lux analytical Ultrahigh, and PSS Suprema Lux 30 Å (PSS, Germany). The separation was performed using 0.3 M lithium acetate buffer as eluent at flow rate 1.0 mL/min, and column temperature was 37°C. Wyatt Dawn 8+ light scattering instrument and Wyatt Optilab T-rex refractive index detector (Wyatt, United States) were used for detection. MW and concentrations were converted from the detector signals by the software ASTRA 7 (Wyatt).

### 2.3. Fecal sample collection and inoculum preparation

Fecal samples were provided by ten human donors with consent. All the donors were self-reported healthy Caucasian males who did not receive pharmaceutical supplementation of pro-, pre-or antibiotics at least 90 days prior to stool collection. The donors were divided into two groups based on their ages, elderly group (*n* = 5, age range 70–75 years old) and young group (*n* = 5, age range 30–35 years old). The studies involving human participants were reviewed and approved by the Ethical Committee of the Capital Region of Denmark (H-15001754; Wiese et al., 2018). Written informed consent to participate in this study was provided by the participants.

The collected fecal samples were processed individually within the same day as described earlier (Wiese et al., 2018). Briefly, fresh feces were mixed with equal weight of phosphate buffered saline (PBS, pH 5.6) containing 20% glycerol, followed by homogenization using a Stomacher 400 (Seward, United Kingdom) at normal speed for 2 min (Wiese et al., 2018). The homogenized fecal slurries were then aliquoted, snap frozen in liquid nitrogen and stored at –60°C until further use. The abovementioned preparation procedures were performed in a vinyl anaerobic chamber (Coy Lab, United States) filled with 5% H_2_, 5% CO_2_ and 90% N_2_ (ALPHAGAZ, Air Liquide, France).

### 2.4. *In vitro* fermentation using CoMiniGut system

Batch fermentations of INU, PEC, PPH and POS with individual fecal inoculum were performed in triplicates using the CoMiniGut system with five parallel reactor units (Wiese et al., 2018). The current study followed the operation protocol of the CoMiniGut model as described (Wiese et al., 2018), with adjustment of several parameters. The homogenized fecal slurries were thawed and further diluted with four volumes of PBS. Each anaerobic reactor unit contained 0.5 mL of PBS-diluted fecal inoculum and 4.5 mL of basal colon medium (2 g/L peptone, 0.5 g/L bile salts, 1 g/L yeast extract, 0.1 g/L NaCl, 0.04 g/L K_2_HPO_4_, 0.04 g/L KH_2_PO_4_, 0.01 g/L MgSO_4_∙7H_2_O, 0.01 g/L CaCl_2_∙2H_2_O, 0.2 g/L NaHCO_3_, 0.005 g/L hemin, 0.001% (v/v) vitamin K1, 0.2% (v/v) Tween 80, 0.5 g/L L-Cysteine HCl; Wiese et al., 2018). The fermentation mixtures had a final concentration of 1% (*w*/*v*) inulin or pectins. The concentrations of the original fecal matter and glycerol were 1 and 0.2%, respectively. Anaerobic conditions in the reactor units were maintained and monitored by placing the Oxoid^™^ AnaeroGen^™^ Compact Sachets and Oxoid^™^ Resazurin Anaerobic Indicator (both from Thermo Fisher Scientific, United States) in the reaction compartments. Fermentations were performed at 37°C, and the pH gradually increased from 5.6 to 6.8 in 24 h, with computer-controlled addition of 1 M sodium hydroxide solution. Fermentations were terminated at 24 h endpoint and samples were stored in aliquots at -60°C until further analysis.

### 2.5. Analysis of short-and branched-chain fatty acids (SCFAs/BCFAs)

Concentrations of SCFAs and BCFAs of the fermentation samples taken at 24 h endpoint were determined by GC–MS as described previously (Wiese et al., 2018, 2021). The fermentation samples were mixed at 1:2 ratio with 0.3 M oxalic acid containing 2 mM 2-ethylbutyrate as internal standard, followed by centrifugation and filtration. One μL of the supernatant was injected into Agilent 7890A GC (Agilent) equipped with a Phenomenex Zebron ZB-WAXplus column (30 m × 250 μm × 0.25 μm, Phenomenex, United States). Agilent 5,973 series MSD mass spectrometer was employed to identity and quantify SCFAs and BCFAs. Selected Ion Monitoring (SIM) mode was used to scan ions with mass–to–charge (*m/z*) ratios of 41, 43, 45, 57, 60, 73, 74, and 84. These *m/z* ions are specific for SCFA and BCFA quantified in this study including acetic acid (*m/z* 43, 45, 60), propionic acid (*m/z* 45, 57, 73, 74), butyric acid (*m/z* 41, 43, 45, 60, 73), isobutyric acid (*m/z* 41, 43, 45, 73), 2-methylbutyric acid (*m/z* 74), valeric acid (*m/z* 41, 45, 60,73), and isovaleric acid (*m/z* 60). Prior to quantification, a calibration curve was constructed for each compound using authentic standards of SCFAs and BCFAs. GC–MS data was analyzed using MATLAB scripts (version R2015A; MathWorks, United States) written by authors. The concentration values were corrected by subtracting the concentrations found in the corresponding blank control (inoculum at baseline) samples.

### 2.6. DNA extraction and 16S rRNA gene amplicon sequencing

DNA was extracted from the fermentation samples taken at 24 h endpoint, and from the blank control samples (inoculum), using the Bead – Beat Micro AX Gravity Kit (A&A Biotechnology, Poland) according to the manufacturer’s instruction. The concentrations of extracted DNA were determined using Qubit^™^ 1X dsDNA High Sensitivity (HS) Assay Kit (Thermo Fisher Scientific) on a Varioskan Flash multimode reader (Thermo Fisher Scientific), followed by a dilution to 1 ng/μL for library preparation.

Library preparation procedures for nanopore based sequencing of V1V3–V8V9 hypervariable regions of 16S rRNA gene amplicon using MinION (Oxford Nanopore Technologies, United Kingdom) were conducted as previously described with several adaptions (Hui et al., 2021). Shortly, in the first round of PCR, 5 μL of the diluted DNA was mixed with 6 μL of nuclease-free water, 12 μL of PCRBIO Ultra Mix (PCR Biosystems Ltd., United Kingdom), and 2 μL of Primers Mix, which contained 5 μM of a unique combination of forward and reverse primers, which sequences were given in Supplementary Table 1 (Kårhus et al., 2022). The mixtures were subjected to the first PCR amplification in a Thermocycler Agilent SureCycler 8800 (Agilent) with the temperature profile as follows: denaturation at 95°C for 5 min; 2 cycles of 95°C for 20 s, 48°C for 30 s, 65°C for 10 s, and 72°C for 45 s; followed by final elongation at 72°C for 4 min. s.

The first PCR products were purified with AMPure XP beads (Beckman Coulter, United States) before the second PCR amplification. The second PCR reaction mixtures contained 11 μL of the first PCR clean products, 12 μL of PCRBIO Ultra Mix, and 2 μL of ONT UMI barcodes at 10 μM that were custom designed for tag encoding multiple samples in one run. The second PCR temperature profile included denaturation at 95°C for 2 min; 33 cycles of 95°C for 20 s, 55°C for 20 s, and 72°C for 40 s; followed by final elongation at 72°C for 4 min. The size of second PCR products (around 1,500 bp) were checked by agarose gel electrophoresis, followed by purification using AMPure XP beads and equimolar pooling. The library preparation was finalized by using the 1D Genomic DNA by Ligation kit (SQK–LSK109, Oxford Nanopore Technologies) according to the manufacturer’s protocol, and was loaded on a R9.4.1 flow cell.

The sequencing data generated by MinION was processed as outlined elsewhere (Hui et al., 2021). MinKnow software v19.06.8,^1^ Guppy v3.2.2 basecalling toolkit (see footnote 1), Porechop v0.2.2,^3^ NanoFilt (qq ≥ 10; read length > 1Kb) were used for data collection, base calling, adapter trimming, demultiplexing and quality correction (Hui et al., 2021). Greengenes (13.8) was used for taxonomy assignment with implementation of parallel_assign_taxonomy_uclust.py in QIIME v1.9.1. Individual Amplicon Sequence Variant (ASV) was treated as individual “seeds.” The reads that were annotated to at least phylum level was included in further analysis. Due to taxonomic reorganization, three features annotated as [*Eubacterium*] *biforme*, *Lactobacillus ruminis*, and *Lactobacillus zeae* were updated manually to *Holdemanella biformis* (De Maesschalck et al., 2014), *Ligilactobacillus ruminis* (Zheng et al., 2020) and *Lacticaseibacillus zeae* (Zheng et al., 2020), respectively.

### 2.7. Data analysis and statistics

Analysis and visualization of SCFAs/BCFAs concentrations and microbiome data was conducted in RStudio v.1.4.1717 integrated in R v.4.1.3. The average SCFAs/BCFAs concentrations of triplicates were taken to check normality and homogeneity of variances using Shapiro–Wilk test and Levene’s test. Comparisons of specific acids among the four substrates were conducted using one-way ANOVA with Tukey’s HSD *post hoc* test. Student’s *t*-test was performed to compare the values between the elderly and the young group. R packages rstatix v.0.7.0, tidy verse v1.3.1, and multcompView v.0.1–8 were used to perform the abovementioned tests.

Microbiome data was imported to R followed by microbial diversity analysis using the package phyloseq v1.38.0 (McMurdie and Holmes, 2013). All the samples were rarefied to an even library depth of 10,000 reads/sample and triplicates were averaged to the nearest integers. One sample using PPH as substrate and fecal inoculum from one elderly donor was discarded due to inadequate library size (< 10,000 counts). Alpha diversity (observed features index and Shannon diversity index) was compared among substrates using one-way ANOVA with Tukey’s HSD *post hoc* test, or between the two age groups using Student’s *t*-test, after checking normality and homogeneity of variances. Beta diversity was evaluated by Jaccard (unweighted) and Bray–Curtis distance matrices. Differences between the substrates as well as between the two age groups were determined by PERMANOVA test using the package vegan v2.5–7 (Dixon, 2003) with *p*-values corrected by Benjamini – Hochberg method. Differentially enriched taxa (summarized at species level) among the substrates were identified by DESeq2 method (Love et al., 2014) with a preset *p*-value lower than 0.05. The taxa occurring at a minimum relative abundance of 1% in at least 10% samples were visualized in a heatmap. Differentially enriched taxa between elderly and young groups were also identified using DESeq2 method (Love et al., 2014). Relative abundance of the responding taxa which fulfilled the abovementioned criteria were compared using Kruskal–Wallis rank sum test. Three features of interest, *s_Faecalibacterium prausnitzii*, *s_Escherichia coli*, and *g_Dorea.s* were also included in the between-age comparison. Pearson’s correlation analysis between centered log-ratio (clr) transformed microbial relative abundance and short-/ branched-chain fatty acid concentrations were performed with Rhea package (Lagkouvardos et al., 2017). Zeros in taxonomic variables were excluded from the correlation analysis. Core taxa were selected with a prevalence cut-off value set as 30%, with at least four pair of observations supporting each calculated correlation. Microbial co-occurrence network based on SPIEC-EASI method was constructed using the unrarefied counts from all the fermentation and fecal inoculum samples (*n* = 129), with R package NetCoMi v.1.0.2 (Peschel et al., 2021). Samples with total reads less than 10,000 were filtered out. Taxa were included in the analysis if prevalence ≥ 30% and mean relative abundance ≥ 0.1%. Other R packages used for visualization included corrplot v0.92 (Wei and Simko, 2021), ggplot2 v.3.3.5 (Wickham, 2016), circlize v.0.4.14 (Gu et al., 2014) and complexheatmap v.2.8.0 (Gu et al., 2016).

## 3. Results

### 3.1. Alpha and beta diversity of microbiota

**Figure 1:**
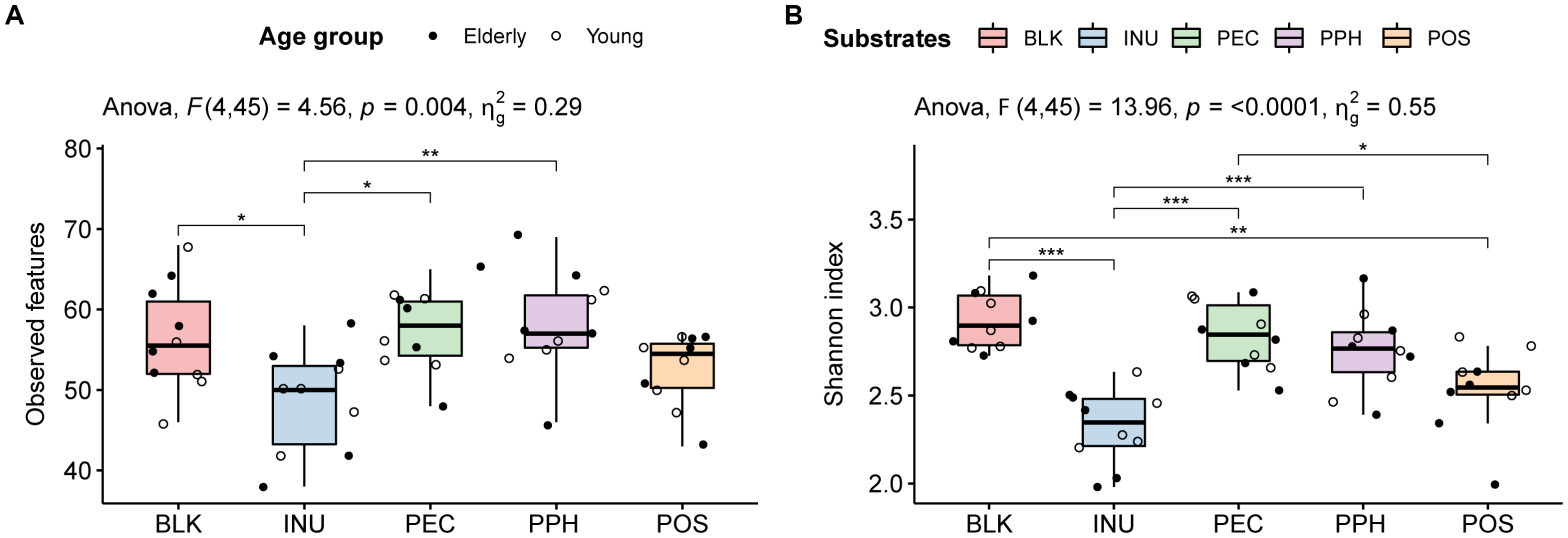
The effect of substrates (BLK, blank control; INU, inulin; PEC, pectin; PPH, partly hydrolyzed pectin; POS, pectin oligosaccharides) and age of fecal inoculum donors (elderly group, *n* = 5; young group, *n* = 5) on the microbial alpha diversity at 24 h end point of CoMiniGut fermentation: observed features summarized at species level **(A)** and Shannon diversity index **(B)**. The labels of ^*^, ^**^, and ^***^ indicate *value of p*s < 0.05, < 0.01, and < 0.001, respectively.

In total, 157 microbial features were summarized at species level. The alpha diversity indices of the fermentations with PEC and PPH were comparable to those of the blank control (inoculum), including observed features (Figure 1A) and Shannon index (Figure 1B). A significant lower Shannon index was observed with POS compared to that with PEC. Fermentation with INU significantly decreased both the observed features and the Shannon diversity index (alpha diversity) compared to the blank control, while POS only had a significantly lower Shannon index than the blank control. The decreased Shannon diversity indices of POS and INU indicated that the two substrates stimulated certain bacterial groups that dominated the corresponding microbial communities. No difference was found between the elderly and young adult groups in terms of alpha diversity (Supplementary Figure 2).

**Figure 2:**
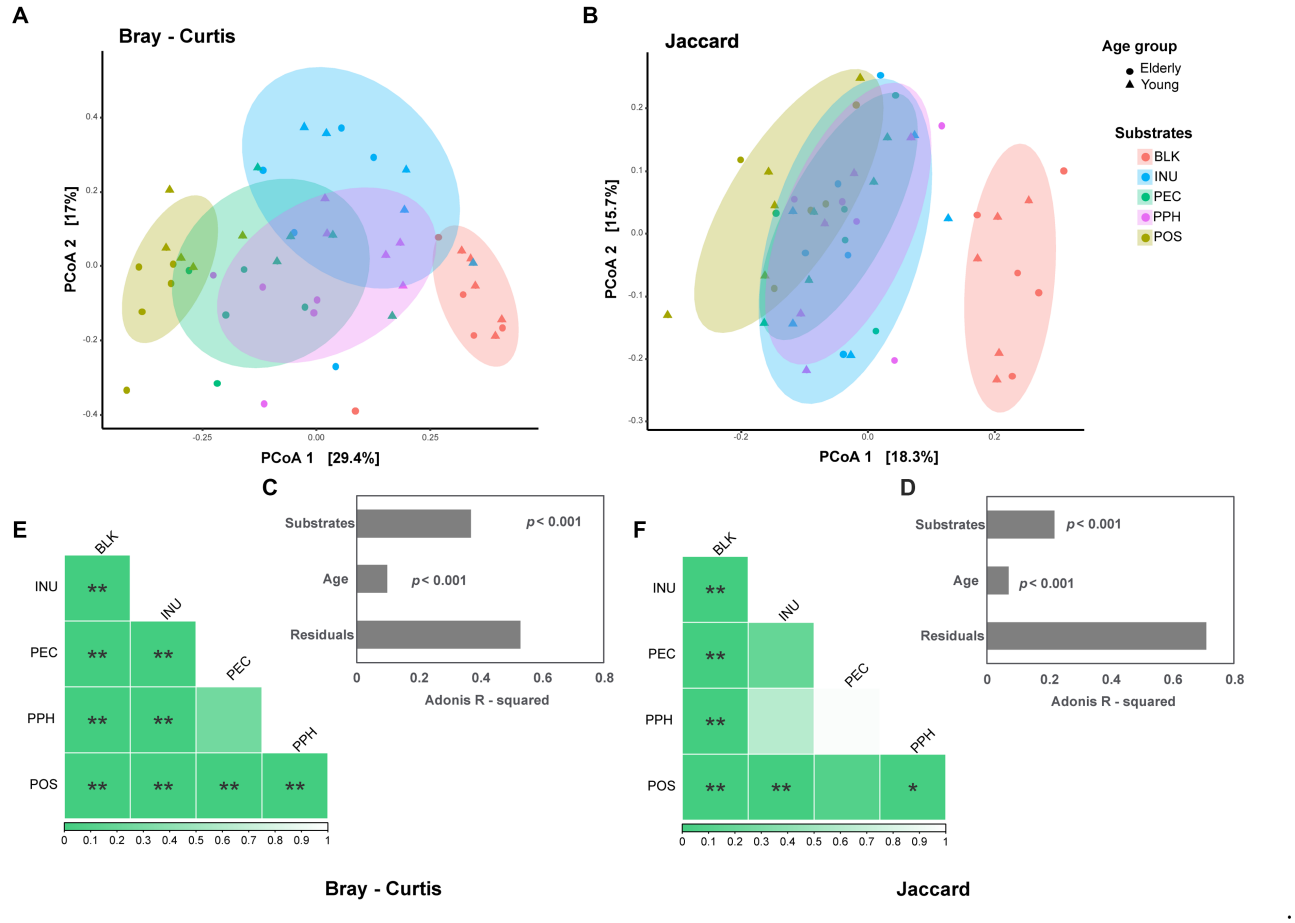
The effect of substrates (BLK, blank control; INU, inulin; PEC, pectin; PPH, partly hydrolyzed pectin; POS, pectin oligosaccharides) and age of fecal inoculum donors (elderly group, *n* = 5; young group, *n* = 5) on the microbial beta diversity at 24 h end point of CoMiniGut fermentation. Principal Coordinates analysis (PCoA) plots based on Bray–Curtis (weighted, **A**) and Jaccard (unweighted, **B**) distance matrices, with their corresponding adonis *R*-squared values listed in **(C)** and **(D)**. The ellipses illustrate the 80% confidential area assuming a multivariable *t*-distribution. Pairwise PERMANOVA tests visualized as heatmaps for Bray–Curtis **(E)** and Jaccard **(F)**, with the *value of p*s indicated by the color codes underneath. The labels of ^*^, ^**^, and indicate *p*-values < 0.05 and < 0.01, respectively.

Principal coordinate analysis (PCoA) of beta diversity was performed based on weighted Bray–Curtis and unweighted Jaccard distance matrices (Figures 2A,B). Both plots showed a shift in community structures of the fermentation samples after 24 h compared to the blank control. Separated clusters corresponding to each substrate were formed in the weighted PCoA, indicating shifts in dominant bacterial groups among the microbial communities. However, the fermentation samples at 24 h clustered closely in the unweighted PCoA, which represented the presence and absence of bacterial groups in the microbial communities. Based on PERMANOVA results, the substrate factor explained 37% of the variances on Bray–Curtis matrix (Figure 2C), compared to 22% of the same factor on Jaccard (Figure 2D). Age had a smaller effect on both matrices than substrate, explaining 10 and 7% on Bray–Curtis and Jaccard, respectively (Figures 2C,D). Differences or similarities in microbial community structures between each substrate and the blank control were further explored by pairwise PERMANOVA (Figures 2E,F). For both weighted and unweighted matrices, all the fermentation samples at 24 h were significantly different from the blank control. PEC and PPH showed comparable community structures, while POS and INU had distinct microbiota profiles compared to PEC and PPH based on the weighted matrix.

### 3.2. Abundances of bacterial groups and co-occurrence network

**Figure 3:**
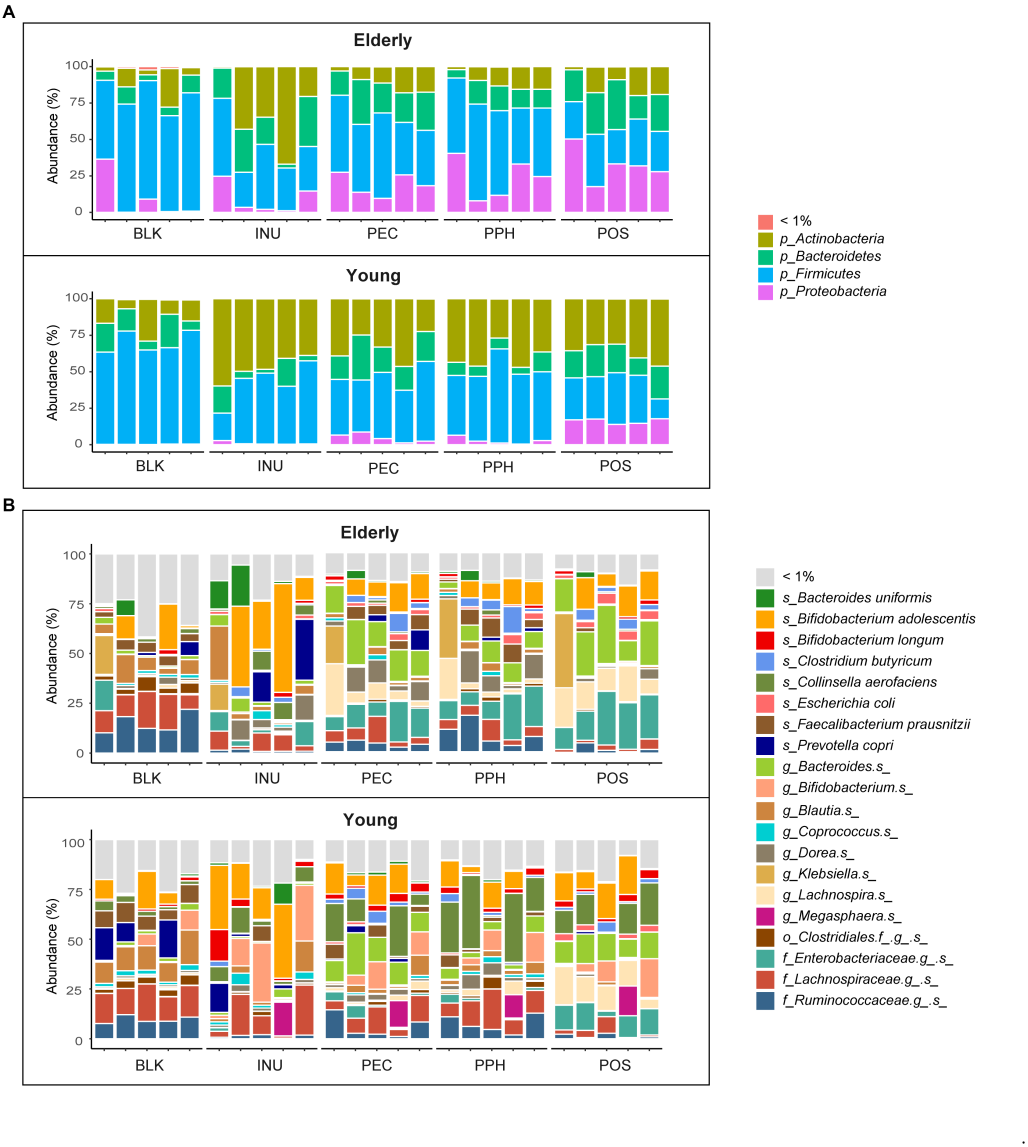
Microbiota composition of blank control (BLK) and at 24 h end point of CoMiniGut fermentation of: INU, inulin; PEC, pectin; PPH, partly hydrolyzed pectin; POS, pectin oligosaccharides. Fecal inoculums used for CoMiniGut fermentation were either from the elderly group (*n* = 5), or the young group (*n* = 5). Relative abundances of detected taxa are summarized at phylum **(A)** and species **(B)** level. Taxa with average relative abundance less than 1% are grouped as “< 1%.”

The average relative abundances of bacterial phyla and genera (abundances ≥ 1%) at 24 h of fermentation as well as the blank control (inoculum) samples are shown in Figures 3A,B. There were 4 phyla and 20 genera with average relative abundances not lower than 1% in all the fermentation samples. Despite the inter-individual differences, Firmicutes was the most abundant phylum in the blank controls in both age groups, dominated by unidentified species from families *Ruminococcaceae* and *Lachnospiraceae*, *Blautia* spp. and *F. prausnitzii*. After 24 h fermentation with INU, the relative abundance of Firmicutes dropped by almost half, while the phylum Actinobacteria increased, represented mainly by *Bifidobacterium adolescentis* in both age groups, and by *Bifidobacterium* spp. in the young adult group only. The decreased relative abundance of Firmicutes was also observed for the pectins, compensated by an increased relative abundance of *Bacteroides* spp., and *B. adolescentis* in both age groups, together with *Collinsella aerofaciens* that was particularly increased in the young adult group. Furthermore, higher levels of phylum Proteobacteria were detected in fermentations with pectins in the elderly group, as well as with POS in the young adult group, which mainly contained an unidentified species from family *Enterobacteriaceae* and a minor fraction of *Escherichia coli*.

**Figure 4:**
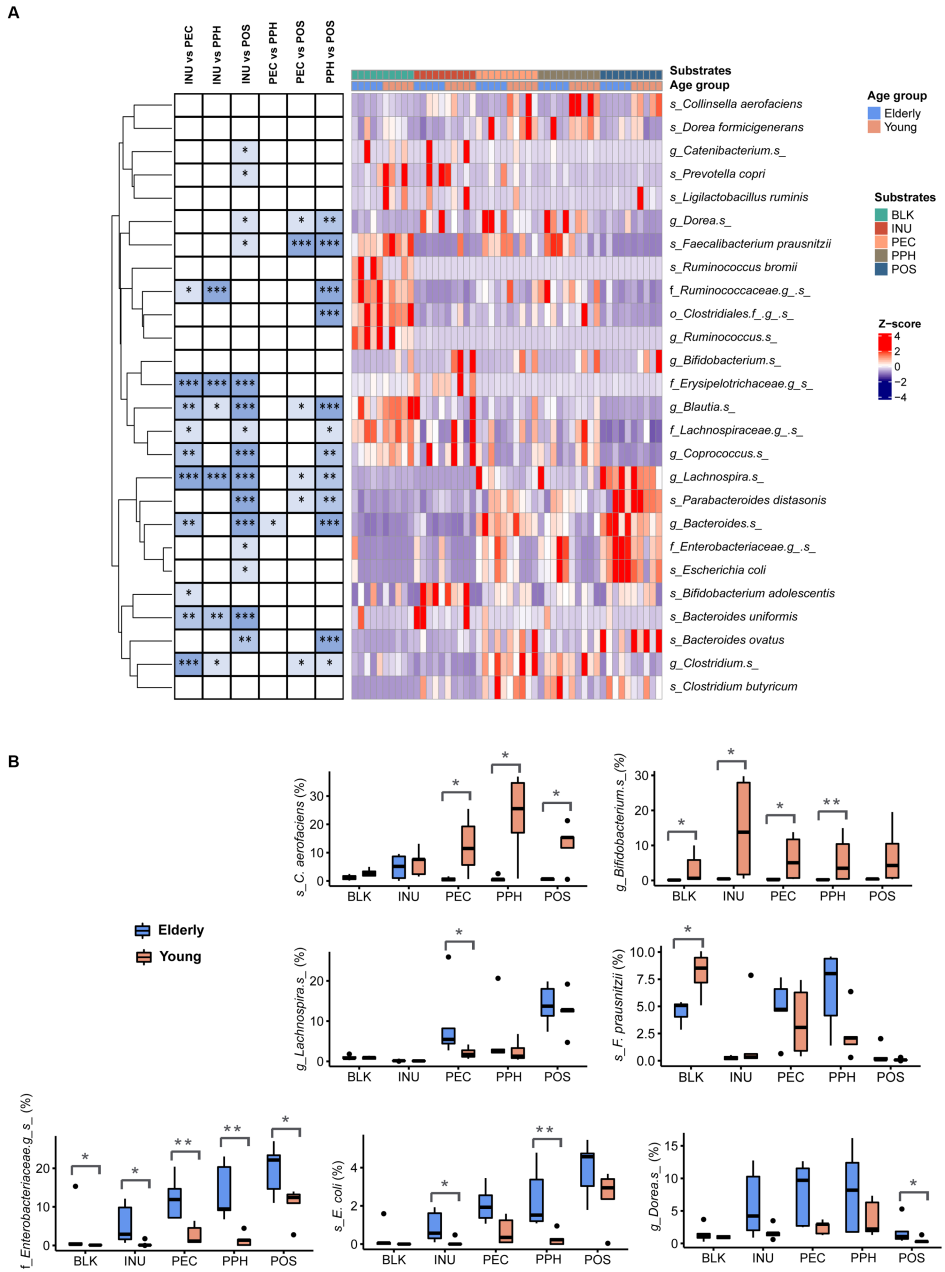
**(A)** Differentially abundant taxa (summarized at species level) from pairwise comparisons between the substrates at 24 h end point of CoMiniGut fermentation, as determined by DESeq 2 analysis. Relative abundances of the taxa were row-wise scaled and visualized in heatmap, where significant *p*-values were indicated with asterisks on the left. **(B)** Relative abundances of selected taxa shown by box plots and compared between elderly group (*n* = 5) and young group (*n* = 5). BLK, blank control; INU, inulin; PEC, pectin; PPH, partly hydrolyzed pectin; POS, pectin oligosaccharides. The labels of ^*^, ^**^, or ^***^ indicate *p*-values < 0.05, < 0.01, or < 0.001, respectively.

Figure 4A visualizes the relative abundances of different taxa between substrates including both age groups. Results of the adapted DESeq2 analysis with *p*-values lower than 0.05 are listed in Supplementary Table 2. In general, PEC and PPH fermentations led to comparable relative abundances of the bacterial groups, except for *Bacteroides* spp., which were more abundant in PEC samples. Fermentations with PEC and PPH favored the growth of *F. prausnitzii*, *Dorea* spp., *Blautia* spp. and *Clostridium* spp., while POS promoted the growth of distinct species, including *Lachnospira* spp., *Parabacteroides distasonis*, *Bacteroides* spp., and *B. ovatus*. Furthermore, fermentation with POS resulted in significantly reduced levels of *Prevotella copri* and *Catenibacterium* spp., along with increased levels of the unidentified species from the *Enterobacteriaceae* family and *E. coli*. Compared to pectins, fermentation with INU was characterized by significantly higher relative abundances of *B. uniformis*, *B. adolescentis*, *Coprococcus* spp., *Blautia* spp. and unidentified species from the families *Lachnospiraceae* and *Erysipelotrichaceae*.

Figure 4B shows the comparison between all the samples from the elderly group to those from the young adult group. Four species with average relative abundances over 1% were significantly different between the groups as based on the DESeq2 analysis, namely *C. aerofaciens*, *Bifidobacterium* spp., *Lachnospira* spp. and the unidentified species from family *Enterobacteriaceae*. The young adult group showed much higher relative abundance of *C. aerofaciens* in response to all the pectins compared to the elderly group. High abundances of *Bifidobacterium* spp. were only detected in the young adult group, especially in fermentations with INU. In contrast, fermentations in the elderly group with almost all the substrates led to increased levels of the unidentified species from family *Enterobacteriaceae* and *E. coli* (Figure 4B). Additionally, the levels of *F. prausnitzii* were significantly lower in the elderly samples compared to the young adult ones in the blank controls (inoculum at baseline; Figure 4B). However, the relative abundances of *F. prausnitzii* became comparable between the elderly and the young groups after 24 h fermentation with PEC and PPH, while it became almost absent with INU and POS.

**Figure 5:**
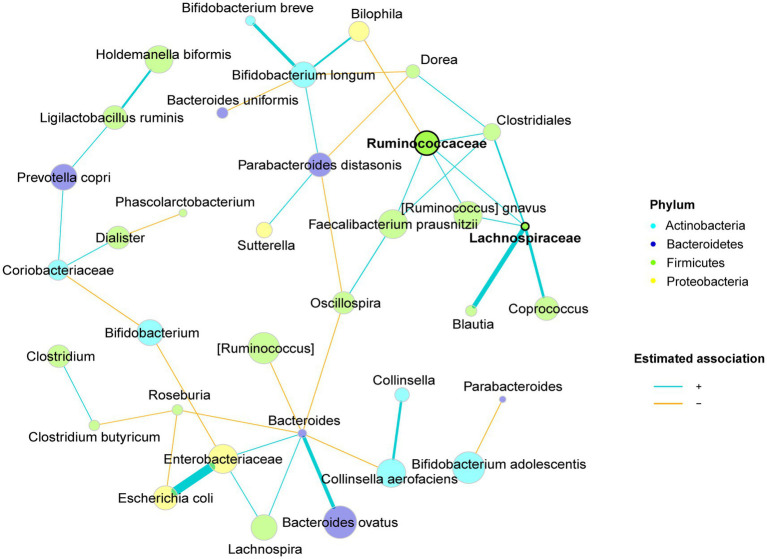
Microbiota co-occurrence network of CoMiniGut fermentation of inulin and pectins using fecal inoculums from either elderly donors (*n* = 5), or young donors (*n* = 5). Taxa are summarized at species level, with phylum level information indicated by node colors. Nodes are defined as hubs based on eigenvector centrality and are highlighted by bold text and borders. Edge thickness and node sizes correspond to similarity values, and centered log-ratio transformed relative abundances of corresponding taxa, respectively.

A microbial co-occurrence network was constructed based on all the samples to explore positive and negative direct connections among bacterial groups (Figure 5). Strong positive associations were commonly found between nodes from the same phylum, for instance, one cluster was formed by several species from the phylum Firmicutes with two unidentified species from families *Ruminococcaceae* and *Lachnospiraceae* as the hubs. Cross-phyla clusters were also observed, e.g., *Bacteroides* spp., *Lachnospira* spp., the unidentified species from family *Enterobacteriaceae* and *E. coli* were co-occurring microbes, which were negatively associated with *Bifidobacterium* spp., *Roseburia* spp., *C. aerofaciens*, *Oscillospira* spp. and *[Ruminococcus]* spp. Despite the phylogenetic similarities, *Bifidobacterium* spp. were seldomly connected, possibly implying low niche overlap and functional diversity within the genus.

### 3.3. Acid production and correlations with microbial taxa

The cumulative production of SCFAs and branched-chain fatty acids (BCFAs) after 24 h fermentation with the four substrates are listed in Table 1. For all the samples, acetic acid, propionic acid, and butyric acid, were present as the dominant acids, while BCFAs (isobutyric acid, isovaleric acid, 2-methylbutyric acid) and valeric acid were detected in minor amounts. Acetic acid was the principal SCFA produced from pectins, followed by butyric acid, and propionic acid produced in least amount. Fermentation with INU led to the highest level of total SCFAs, butyric acid and propionic acid within the young adult group. Interestingly, the SCFAs levels became statistically insignificant between INU and the pectins after 24 h fermentation by the elderly microbiota. When comparing the two age groups, elderly microbiota showed lower capability of utilizing inulin for butyric acid production, but no difference utilizing pectins, compared to young adults. The fact that microbiota of the young adult group generated significantly higher concentrations of butyric acid than that of the elderly with INU indicated that the bifidogenic effect of inulin is age dependent. Furthermore, no significant differences were identified on BCFAs and valeric acid production among substrates or between age groups (*p*-values listed in Supplementary Table 3).

**Table 1:** Cumulative production of short-and branched-chain fatty acids (SCFAs/BCFAs) in mmol or μmol per gram of substrates (INU, inulin; PEC, pectin; PPH, partly hydrolyzed pectin; POS, pectin oligosaccharides) at 24 h end point of CoMiniGut fermentation^#^.

Organic acids	Substrates				
	INU	PEC	PPH	POS	*p*-Value^*^
ELDERLY					
*Major SCFAs (mmol/g)*					
Acetic acid	1.86 (0.26)	2.34 (0.28)	2.32 (0.37)	2.14 (0.26)	0.07
Propionic acid	1.11 (0.88)	0.76 (0.09)	0.72 (0.11)	0.66 (0.28)	0.45
Butyric acid	1.60 (0.75)^†^	1.04 (0.61)	1.15 (0.63)	0.98 (0.53)	0.44
Total	4.56 (1.12)^†^	4.15 (0.3)^†^	4.2 (0.77)	3.78 (0.65)	0.48
*BCFAs (μmol/g)*					
Isobutyric acid	9.17 (1.40)	10.87 (5.49)	15.78 (7.24)	11.21 (13.89)	0.29
Isovaleric acid	3.68 (2.76)	7.95 (4.3)	9.34 (6.16)	8.95 (13.23)	0.26
2-Methylbutyric acid	2.98 (1.75)	4.99 (2.82)	6.67 (5.17)	6.26 (8.46)	0.51
Total	15.83 (5.09)	23.8 (12.22)	31.8 (17.71)	26.43 (35.55)	0.36
*Other (μmol/g)*					
Valeric acid	33.61 (34.65)	13.33 (12.61)	19.38 (17.38)	2.93 (1.89)	0.16
YOUNG					
*Major SCFAs (mmol/g)*					
Acetic acid	1.91 (0.73)	2.31 (0.42)	2.27 (0.47)	1.89 (0.38)	0.44
Propionic acid	1.3 (0.43)^a^	0.78 (0.27)^ab^	0.82 (0.37)^ab^	0.6 (0.2)^b^	**0.02**
Butyric acid	2.84 (0.76)^a^	1.47 (0.35)^b^	1.69 (0.49)^b^	1.45 (0.48)^b^	**0.002**
Total	6.05 (0.5)^a^	4.56 (0.24)^b^	4.78 (0.25)^b^	3.94 (0.22)^c^	**<0.001**
*BCFAs (μmol/g)*					
Isobutyric acid	16.43 (8.8)	21.89 (13.48)	22.41 (5.41)	11.61 (8.51)	0.27
Isovaleric acid	6.37 (4.07)	15.62 (12.51)	13.48 (4.18)	8 (7.56)	0.24
2-Methylbutyric acid	5.93 (4.45)	10.04 (7.84)	9.54 (3.76)	6.05 (5)	0.51
Total	28.73 (16.38)	47.56 (33.53)	45.42 (11.89)	25.66 (20.16)	0.31
*Other (μmol/g)*					
Valeric acid	14.65 (19.59)	42.84 (76.64)	74.62 (125.54)	50.2 (105.93)	0.65

# Values with different superscript letters were significantly different from each other in the same row, with their averages ranked from higher to lower following the order a to c.

* Difference in specific acid concentrations among the four substrates within the same age group.

† Significantly different concentrations of the same acids between elderly and young on the same substrates (*p* < 0.05).

The results are shown as means (standard deviations).

**Figure 6:**
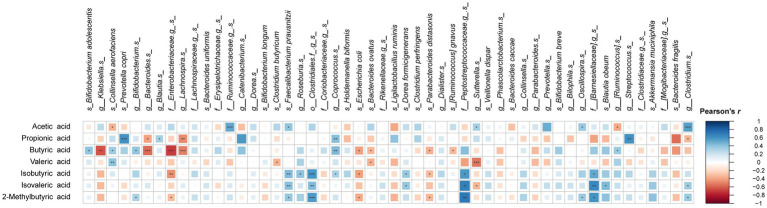
Pearson’s correlation between relative abundances of microbial taxa (summarized at species level) and short-and branched-chain fatty acid concentrations at 24 h end point of CoMiniGut fermentation. The areas of colored squares correspond to the absolute values of correlation coefficients (*r*). The labels of ^*^, ^**^, or ^***^ indicate *p*-values < 0.05, < 0.01, or < 0.001, respectively.

Pearson’s correlation between cumulative acid production and microbial taxa at species level are summarized in Figure 6. Interestingly, butyric acid, propionic and valeric acid showed similar correlations with specific species, whereas acetic acid often showed opposite correlations with the same species. Positive correlation with acetic acid levels were observed for *F. prausnitzii* and *Clostridium* spp., which were highly abundant species in PEC and PPH fermentations. The species favored by POS, such as *Lachnospira* spp., *P. distasonis*, *Bacteroides* spp., and *B. ovatus*, correlated negatively with butyric and/or propionic acid production. Abundances of *B. adolescentis*, *Coprococcus* spp., *Blautia* spp. and *P. copri*, which were enriched species in INU fermentations, positively correlated with the levels of butyric and/or propionic acid. Finally, *C. aerofaciens* and *Bifidobacterium* spp., which were found significantly abundant in the young adult group, correlated positively with butyric acid. *E. coli* and the unidentified species from family *Enterobacteriaceae*, specifically found in the elderly group, correlated negatively with butyric acid.

## 4. Discussion

### 4.1. Effects of pectins on the gut microbiota in relation to structural differences

PEC and its derivatives, PPH and POS, were included in the same experimental setup to study the impact of MW of pectin on their fermentation by gut microbiota. Fermentation of PPH resulted in similar fermentation profiles as the parent pectin polysaccharide PEC, in terms of both microbial diversity and relative abundances of bacterial groups. Both substrates selectively promoted the abundances of *F. prausnitzii*, *Dorea* spp., *Blautia* spp. and *Clostridium* spp. after 24 h *in vitro* fermentation, probably due to their capacity to degrade the polysaccharide backbones of pectin (Lopez-Siles et al., 2012). *F. prausnitzii* is one important primary pectin-degrader, whose activity releases sugar units from the pectin backbone for further consumption by secondary metabolizers (Elshahed et al., 2021). In addition to being one of the most abundant species present in the gut microbiota of healthy humans, *F. prausnitzii* is considered a beneficial bacterium to treat gut inflammation (Lopez-Siles et al., 2012; Miquel et al., 2013; Heinken et al., 2014). The ability of PEC and PPH to promote the growth of *F. prausnitzii* is promising for the elderly population, who are vulnerable to age-related inflammation (An et al., 2018; Míguez et al., 2020). In accordance with the present findings, our previous *in vitro* study exploring the effects of pectins (Larsen et al., 2019), found that the abundances of *F. prausnitzii*, together with family *Ruminococcaceae*, were most stimulated by high methylated lemon pectin. Furthermore, we found a positive correlation between acetic acid production and *F. prausnitzii*, which agreed with a previous study showing that *in vitro* growth of *F. prausnitzii* was stimulated by presence of acetate in the medium (Heinken et al., 2014).

Fermentation with POS, having the lowest MW, resulted in the lowest Shannon diversity index and a significantly different beta diversity based on Bray-Curtis compared to PEC and PPH. The Shannon diversity index estimates richness and evenness of a microbial community, and a lower value is related to lower stability or less homeostasis of the human microbiome (An et al., 2018). Although several previous *in vitro* studies have reported an increased bifidogenic effect of lower MW pectins and POS (Olano-Martin et al., 2002; Gómez et al., 2016; Ferreira-Lazarte et al., 2018), we did not observe such effects in fermentations with POS in the current set-up. The lower bifidogenic effects of POS from our study than the previous studies could be due to structural differences of POS since they were derived from different parent pectins (Pascale et al., 2022). The species that were promoted by POS in the current study included *Lachnospira* spp., *P. distasonis*, *Bacteroides* spp., and *B. ovatus*. In contrast to PEC and PPH, the beneficial species *F. prausnitzii*, was almost depleted after 24 h fermentation with POS. Microbial utilization of large pectin polysaccharides requires extracellular breakdown of the backbone by pectinolytic activities before intracellular consumption (Elshahed et al., 2021). We speculate that the capability of *F. prausnitzii* to degrade the polymer backbone of PEC and PPH might account for its specificity to pectin polysaccharides instead of oligosaccharides.

Another reason for the differences observed in microbiota profiles could be a slower fermentation of PEC and PPH compared to POS, due to the time needed to degrade the polymer structures of PEC and PPH. The relatively faster fermentation of POS by the primary pectin-degraders could be already followed by enrichment of the secondary metabolizers by cross-feeding interactions within 24 h (Bang et al., 2018), leading to different microbiota composition compared to PEC and PPH. This hypothesis of slow fermentation is supported by one *in vitro* study that showed a trend from rise to decline of relative abundances of *F. prausnitzii* during fermentation with methylated pectin oligosaccharides (Onumpai et al., 2011). A slow fermentation of pectins may be more promising in the sense that the beneficial saccharolytic activities could be prolonged to the distal colon (Tingirikari, 2018). Nevertheless, the SCFAs and BCFAs profiles of all three pectins were comparable in this study, implying functional redundancy of the gut microbiome on pectin fermentation pathways (Reichardt et al., 2018).

### 4.2. Effects of pectins on the gut microbiota in relation to donor age

Donors’ age was another factor affecting the fermentation profiles. The fecal microbiota of the elderly group in the blank control (inoculum) had lower levels of *F. prausnitzii* and *Bifidobacterium* spp., while the family *Enterobacteriaceae* was enriched, compared to the young adult group. After 24 h fermentation with pectins, the phylum Bacteroidetes, which is often equipped with pectinolytic enzymes, was increased relatively more in the elderly group. In contrast, the young adult group exhibited a higher enrichment of the phylum Actinobacteria, which generally includes secondary metabolizers instead of primary pectin-degraders (Chung et al., 2017; Tingirikari, 2018). The different microbial profiles at 24 h of fermentation between the two age groups may indicate a slower fermentation of pectins by elderly gut microbiota, as the elderly microbiota were still dominated by the primary pectin-degraders.

Interestingly, the relative abundances of *F. prausnitzii*, although lower in the elderly inoculum, were equally abundant in both age groups, after 24 h fermentation with PEC and PPH. Several other bacterial taxa were found to be associated with donor age. Among them, *C. aerofaciens*, which levels were comparably low in the inoculum of both age groups, was promoted by all pectins in the young adult group, while almost absent in the elderly group. In contrast, species from family *Enterobacteriaceae* and *E. coli* were much more abundant in the elderly group at the end of fermentation. In accordance, we found a negative correlation between *C. aerofaciens* and the cluster comprising *Enterobacteriaceae* and *E. coli* in the microbial network. These findings are in line with those of Miguez et al., who reported an increased growth of *F. prausnitzii* and near absence of genus *Collinsella* in POS *in vitro* fermentations with a pooled elderly fecal inoculum (Míguez et al., 2020). The species *C. aerofaciens* showed a positive correlation with butyric acid production, and in accordance with our results, Qin et al. (2019) reported it as a butyrate-producing strain. Several studies found higher prevalence of *C. aerofaciens* in healthy controls, compared to patients with cystic fibrosis or inflammatory bowel disease (IBD; Miragoli et al., 2017; Malham et al., 2019; Quagliariello et al., 2020), suggesting its association with a healthy microbiome. The family *Enterobacteriaceae*, including *E. coli*, have been reported to possess genes encoding pectinases, however, *in vitro* studies demonstrated non-promoting and anti-adhesive properties of pectins against pathogenic *E. coli* strains in human cell models (Di et al., 2017; Prandi et al., 2018).

Unlike other *Bifidobacterium* spp., *B. adolescentis* was equally stimulated by pectins in both age groups, and it was isolated from other species in the microbial network. The high relative abundance of *B. adolescentis* among elderly has also been observed in previous studies (Míguez et al., 2020). A recent review has listed evidence showing that *B. adolescentis* has a potential key role in modulating gut-brain axis interactions by producing gamma aminobutyric acid (GABA), which inhibits anxiety and depression (Barrett et al., 2012; Sharma et al., 2021). Furthermore, *B. adolescentis* was found to be anti-inflammatory and had an anti-viral effect against Noroviruses (Li et al., 2016; Sharma et al., 2021).

Despite the different microbiota composition observed between the two age groups, their microbial richness and evenness were similar. The 24 h cumulative production of SCFAs and BCFAs from the pectins were also comparable, except for a slightly lower total SCFAs level observed in fermentations with PEC. This finding further confirmed the high level of functional redundancy of pectin fermentation for SCFA production by microbes in the human gut levels out the age effect.

### 4.3. Comparative effects of pectins and inulin on the gut microbiota composition and SCFA production

In the present study, we found the well-known bifidogenic and butyrogenic effects of inulin drastically reduced in fermentations with the gut microbiota of the elderly population, in an *in vitro* experimental setup. The reduced bifidogenic effect of inulin with elderly gut microbiota has also been reported in literature. An et al. (2021) noticed less consistent increase of *Bifidobacterium* spp. abundances with fermentation of traditional bifidogenic ingredients, including inulin, by pre-frail elderly compared to adult controls, which the authors suggested was due to a reduced efficiency in carbohydrate degradation of the pre-frail elderly gut microbiota. Furthermore, they found that the lower level of *Bifidobacterium* spp. allowed utilization of inulin by other bacterial groups, e.g., *Blautia* spp. (An et al., 2021). Some human intervention studies found an increase in bifidobacterial prevalence with inulin supplementation (Marteau et al., 2011; Kiewiet et al., 2021). However, these intervention studies only included elderly participants, without a control group of younger adults while it is therefore difficult to compare the two age groups. In the current study, enriched *Enterobacteriaceae* family and *E.coli* were detected in elderly group with inulin, which had negative correlations with butyrate production, as well as negative relationships with *Bifidobacterium* spp. Production of SCFAs from carbohydrates is believed to be highly reproducible despite inter-individual variations in gut microbiota compositions (Reichardt et al., 2018). The drastic reduction of the butyrogenic effect of inulin observed in the current study indicates a loss of functional genes in the elderly gut microbiota which encodes SCFA synthesis, and possibly a lower level of functional redundancy of the gut microbiota in utilizing inulin, compared to that of pectins. *F. prausnitzii* was reported to be another degrader of inulin in the human gut in literature (Park et al., 2022). However, the selective stimulating effects of inulin on *F. prausnitzii* was not observed in the current study. The inulin-utilizing capabilities varied among different strains of *F. prausnitzii* (Lopez-Siles et al., 2017). The strains of *F. prausnitzii* found in the current study could not be those efficient degraders of inulin. As limited by the sequencing technique used, we were not able to study the gut microbes at strain level.

Overall, both inulin and pectins led to distinct fermentation profiles for both the elderly and the young adult groups. In general, effects of pectins on the gut microbiota were more specific and less dependent on the initial microbiota of the fecal inoculum compared to inulin, confirming the results obtained by a previous study (Cantu-Jungles et al., 2021). Pectin fermentations were characterized with enriched *Bacteroides* spp., *P. distasonis*, *Lachnospira* spp. and *Clostridium* spp., with a specific promotion of *F. prausnitzii* by pectin polymer structures, as well as a specific increase in *C. aerofaciens* found in gut microbiota from young donors. Out of the aforementioned species, *Bacteroides* spp., *P. distasonis*, *Lachnospira* spp. and *F. prausnitzii* are primary pectin-degraders equipped with pectinases, while the remaining ones would utilize released sugars for metabolite conversion such as SCFA production (Elshahed et al., 2021). *Bifidobacterium* spp. are known to encode enzymes like β-fructosidase for inulin degradation (Gibson et al., 2017). However, this genus is not able to degrade pectins and may function as secondary metabolizers via cross-feeding interactions (Yang et al., 2013). Although *Bifidobacterium* is not a butyrate-producer (Sarbini and Rastall, 2011), its abundance positively correlated with butyrate levels in the current study, indicating the existence of cross-feeding interactions between *Bifidobacterium* and butyrate-producing microbes. As a result, we found that inulin fermentations generated more butyric acid by the young adult group compared to pectins, which generated mainly acetic acid, and this finding is in accordance with previous *in vitro* studies (Johnson et al., 2015; Chung et al., 2016; Bang et al., 2018; Chung et al., 2019; Yu et al., 2020). Clinical trials confirming the consistently induced acetate levels by pectin supplementation are so far rare. Acetic acid might play a role in protecting against pathogens and inflammation (Míguez et al., 2020), which is often associated with aging.

## 5. Conclusion

By using an *in vitro* colonic fermentation set-up, we showed how the molecular sizes of HM citrus pectins impacted their colonic fermentation by the human gut microbiota. Pectins of large and medium-size molecules led to more similar fermentation profiles compared to pectin oligosaccharides, in terms of the gut microbiota compositions and SCFA productions. The commonly known beneficial gut microbe, *F. prausnitzii*, was specifically stimulated by fermentation of pectin polysaccharides (both large and medium sizes) instead of pectin oligosaccharides. As to the SCFA production, acetic acid was the main SCFA product from all the pectins. Fermentations with inulin led to microbial profiles different from those of the pectin substrates, in particular higher increase in *Bifidobacterium* spp. and production of butyric acid were seen. Donor age also influenced the fermentation results of both inulin and pectins. However, in contrast to the obviously reduced bifidogenic and butyrogenic effects of inulin on the elderly gut microbiota, fermentations of pectins were less dependent on the donor age. Therefore, HM citrus pectins of high and medium molecular sizes showed the most potential to improve age-related dysbiosis of the elderly gut microbiota. Although an *in vitro* experimental setup is an efficient screening tool to evaluate and compare the potential effects of different substrates on the human gut microbiota, the observed effects should be further confirmed by human intervention studies.

## Data availability statement

The data presented in the study are deposited in the NCBI database under BioProject PRJNA985075, https://www.ncbi.nlm.nih.gov/bioproject/PRJNA985075.

## Ethics statement

The studies involving human participants were reviewed and approved by the Ethical Committee of the Capital Region of Denmark (H-15001754; Wiese et al., 2018). Written informed consent to participate in this study was provided by the participants.

## Author contributions

LJ and NL: conceptualization and supervision. FG: experiments, data analysis, and writing—draft preparation. BK: SCFA analysis. NL, NP, FR, SP, BK, and LJ: writing—review and editing. All authors contributed to the article and approved the submitted version.

## Funding

This project was financially supported by the Innovation Fund Denmark (Case Number 0154-00018A).

## Conflict of interest

FG, NP, FR, and SP are employees of CP Kelco Inc., a producer of commercial pectin.

The remaining authors declare that the research was conducted in the absence of any commercial or financial relationships that could be construed as a potential conflict of interest.

## Publisher’s note

All claims expressed in this article are solely those of the authors and do not necessarily represent those of their affiliated organizations, or those of the publisher, the editors and the reviewers. Any product that may be evaluated in this article, or claim that may be made by its manufacturer, is not guaranteed or endorsed by the publisher.

## Footnotes

^1^ https://nanoporetech.com

^3^ https://github.com/rrwick/Porechop
