# Supplementary material for: Age-related effects on the modulation of gut microbiota by pectins and their derivatives: an in vitro study
Source: Front Microbiol. 2023 Jul 5;14:1207837. doi: 10.3389/fmicb.2023.1207837 (PMC10354267; doi:10.3389/fmicb.2023.1207837)
Supplement: Supplementary file 1 [file Data_Sheet_1.docx]

Supplementary Material

Age-Related Effects on the Modulation of Gut Microbiota by Pectins and Their Derivatives: An *In Vitro* Study

Fangjie Gu*, Nadja Larsen, Nélida Pascale, Sune Allan Petersen, Bekzod Khakimov, Frederique Respondek and Lene Jespersen

*** Correspondence:** Fangjie Gu: fangjie.gu@food.ku.dk

# Supplementary Figures and Tables

## Supplementary Figures


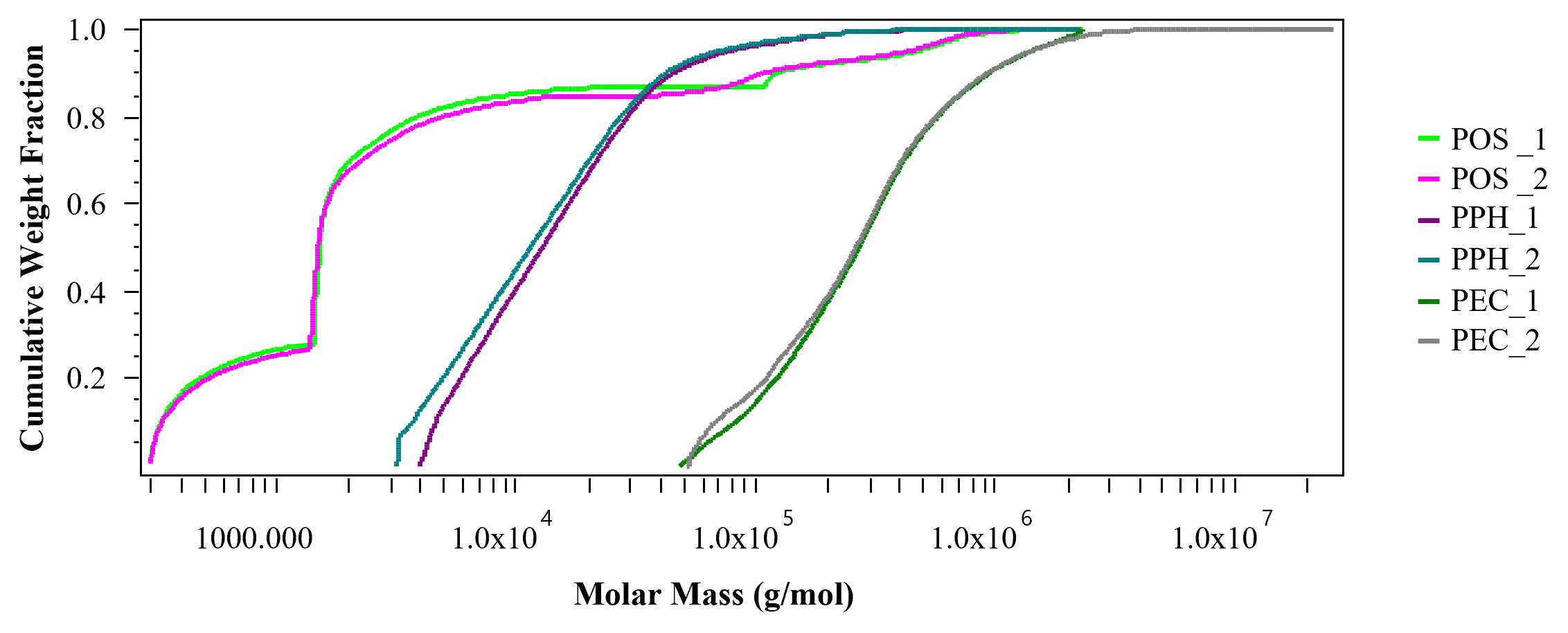


**Supplementary Figure 1.** Molecular weight distribution of the three pectic substrates: PEC, pectin; PPH, partly hydrolyzed pectin; POS, pectin oligosaccharide. The numbers in the legend mean each substrate was analyzed in duplicates.


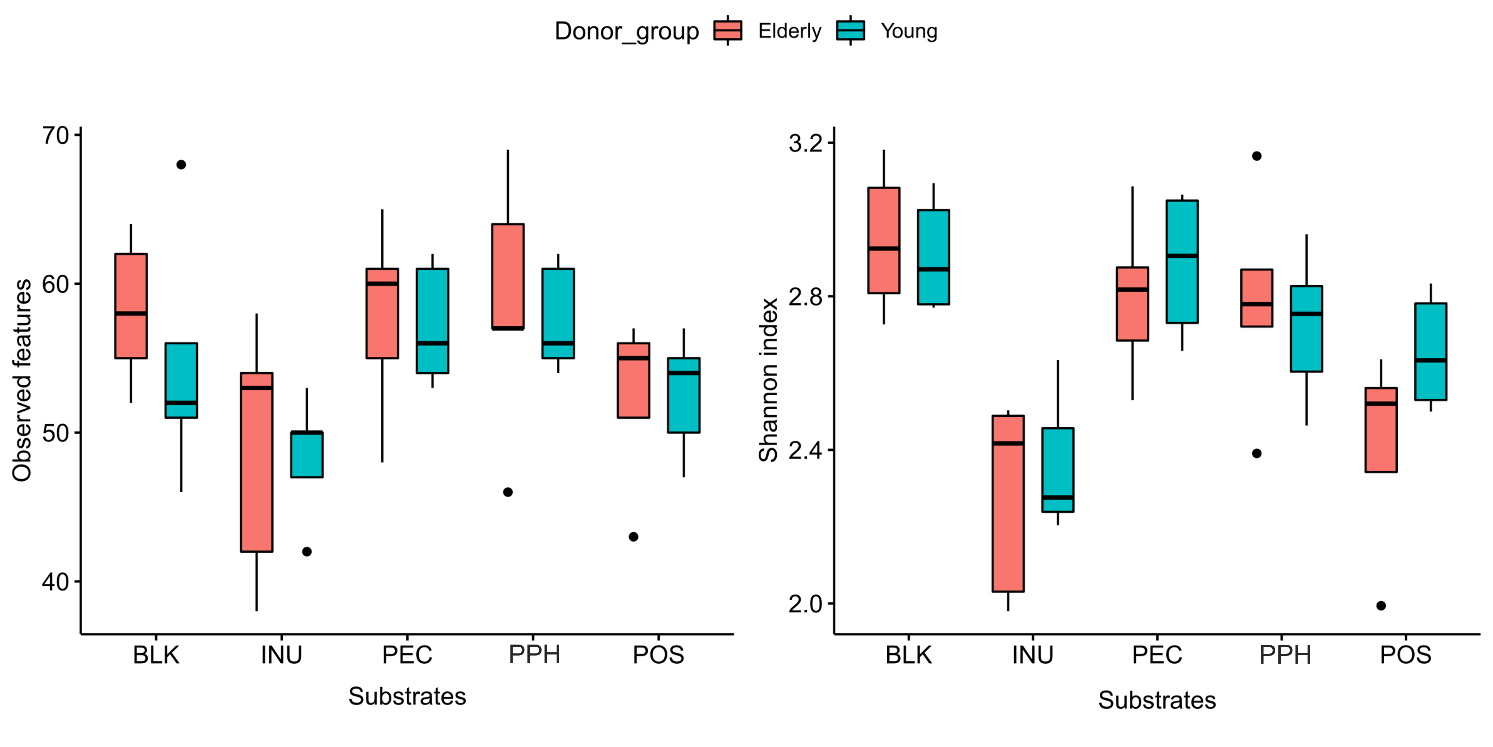


**Supplementary Figure 2**. Microbial alpha diversity estimates, including observed features summarized at species level, and Shannon diversity index, were compared between elderly (n = 5) and younger adults (n = 5) donor groups. BLK, blank control; INU, inulin; PEC, pectin; PPH, partly hydrolyzed pectin; POS, pectin oligosaccharides.

## Supplementary Tables

**Supplementary Table 1**. Primer sequences used for near-full length 16S rRNA gene amplicon sequencing. The NNN-stretch represents unique molecular identifier (UMI) -barcodes, while the nucleotides in the 5’-end of the primer represents adapter sequences for the second PCR.

| **Primers** | **Primer Sequence** |
| --- | --- |
| UMI_338Fa | 5’- GTCTCGTGGGCTCGG- NNNNNNNNNNNNNNN - ACWCCTACGGGWGGCAGCAG-3’ |
| UMI_338Fb | 5’- GTCTCGTGGGCTCGG- NNNNNNNNNNNNNNN - GACTCCTACGGGAGGCWGCAG-3’ |
| UMI_27Fa | 5’- GTCTCGTGGGCTCGG- NNNNNNNNNNNNNNN - AGAGTTTGATYMTGGCTYAG-3’ |
| UMI_27Fb | 5’- GTCTCGTGGGCTCGG- NNNNNNNNNNNNNNN - AGGGTTCGATTCTGGCTCAG-3’ |
| UMI_1540R | 5’- GTCTCGTGGGCTCGG- NNNNNNNNNNNNNNN - TACGGYTACCTTGTTACGACT-3’ |
| UMI_1391R | 5’- GTCTCGTGGGCTCGG- NNNNNNNNNNNNNNN - GACGGGCGGTGTGTRCA-3’ |

**Supplementary Table 2**. Differentially abundant taxa (summarized at species level) among the substrates at 24 h end point of CoMiniGut fermentation, as determined by DESeq 2. The significant *p*-values generated from pairwise comparisons were listed in the table. BLK, blank control; INU, inulin; PEC, pectin; PPH, partly hydrolyzed pectin; POS, pectin oligosaccharides. N.S. means no significant difference was found.

| **Species level taxa** | **BLK vs INU** | **BLK vs PEC** | **BLK vs PPH** | **BLK vs POS** | **INU vs PEC** | **INU vs PPH** | **INU vs POS** | **PEC vs PPH** | **PEC vs POS** | **PPH vs POS** |
| --- | --- | --- | --- | --- | --- | --- | --- | --- | --- | --- |
| *s_Collinsella aerofaciens* | 0.0039 | N.S. | 0.0103 | 0.0147 | N.S. | N.S. | N.S. | N.S. | N.S. | N.S. |
| *s_Dorea formicigenerans* | N.S. | 0.0185 | N.S. | N.S. | N.S. | N.S. | N.S. | N.S. | N.S. | N.S. |
| *g_Catenibacterium.s_* | N.S. | N.S. | N.S. | N.S. | N.S. | N.S. | 0.0254 | N.S. | N.S. | N.S. |
| *s_Prevotella copri* | N.S. | N.S. | N.S. | 0.0089 | N.S. | N.S. | 0.0183 | N.S. | N.S. | N.S. |
| *s_Lactobacillus ruminis* | N.S. | N.S. | 0.0450 | N.S. | N.S. | N.S. | N.S. | N.S. | N.S. | N.S. |
| *g_Dorea.s_* | 0.0006 | 0.0001 | 0.0002 | N.S. | N.S. | N.S. | 0.0141 | N.S. | 0.0317 | 0.0092 |
| *s_Faecalibacterium prausnitzii* | 3.63e-08 | N.S. | N.S. | 3.77e-07 | N.S. | N.S. | 0.0258 | N.S. | 0.0003 | 2.55e-05 |
| *s_Ruminococcus bromii* | 0.0004 | 9.66e-06 | 0.0056 | 1.06e-05 | N.S. | N.S. | N.S. | N.S. | N.S. | N.S. |
| *f_Ruminococcaceae.g_.s_* | 1.15e-11 | 0.0024 | N.S. | 2.62e-06 | 0.0368 | 0.0007 | N.S. | N.S. | N.S. | 0.0003 |
| *o_Clostridiales.f_.g_.s_* | 0.0042 | 1.49e-08 | 0.0287 | 2.66e-09 | N.S. | N.S. | N.S. | N.S. | N.S. | 0.0004 |
| *g_Ruminococcus.s_* | 5.43e-08 | 3.78e-08 | 5.28e-07 | 7.69e-23 | N.S. | N.S. | N.S. | N.S. | N.S. | N.S. |
| *g_Bifidobacterium.s_* | 0.0499 | N.S. | N.S. | N.S. | N.S. | N.S. | N.S. | N.S. | N.S. | N.S. |
| *f_Erysipelotrichaceae.g_.s_* | 0.0012 | 0.0003 | 0.0112 | 0.0258 | 1.43e-07 | 6.35e-06 | 0.0001 | N.S. | N.S. | N.S. |
| *g_ Blautia.s_* | N.S. | 9.95e-05 | 2.28e-05 | 4.13e-15 | 0.0043 | 0.0109 | 4.92e-06 | N.S. | 0.0104 | 3.63e-05 |
| *f_Lachnospiraceae.g_.s_* | N.S. | 0.0052 | N.S. | 4.4e-05 | 0.0481 | N.S. | 0.0191 | N.S. | N.S. | 0.0367 |
| *g_Coprococcus.s_* | N.S. | N.S. | N.S. | 6.69e-06 | 0.0048 | N.S. | 6.43e-05 | N.S. | N.S. | 0.0017 |
| *g_ Lachnospira.s_* | 2.3e-06 | 9.07e-06 | 0.0008 | 1.64e-25 | 4.94e-14 | 3.26e-10 | 1.19e-49 | N.S. | 0.0499 | 0.0093 |
| *s_Parabacteroides distasonis* | 0.0499 | 0.0002 | 0.0032 | 2.66e-09 | N.S. | N.S. | 0.0004 | N.S. | 0.0499 | 0.0092 |
| *g_Bacteroides.s_* | 0.0319 | 3.64e-17 | 2.28e-05 | 2.25e-18 | 0.0014 | N.S. | 5.6e-06 | 0.0103 | N.S. | 2.55e-05 |
| *f_ Enterobacteriaceae.g_.s_* | 1.96e-05 | 0.0495 | N.S. | 1.86e-06 | N.S. | N.S. | 0.0364 | N.S. | N.S. | N.S. |
| *s_Escherichia coli* | N.S. | N.S. | N.S. | 0.0035 | N.S. | N.S. | 0.0490 | N.S. | N.S. | N.S. |
| *s_Bifidobacterium adolescentis* | 0.0279 | N.S. | N.S. | N.S. | 0.0481 | N.S. | N.S. | N.S. | N.S. | N.S. |
| *s_Bacteroides uniformis* | 0.0061 | 0.0038 | N.S. | N.S. | 0.0015 | 0.0013 | 0.0010 | N.S. | N.S. | N.S. |
| *s_Bacteroides ovatus* | 0.0380 | 0.0003 | 0.0305 | 7.3e-06 | N.S. | N.S. | 0.0034 | N.S. | N.S. | 0.0003 |
| *g_ Clostridium.s_* | N.S. | 0.0002 | 0.0102 | N.S. | 0.0001 | 0.0109 | N.S. | N.S. | 0.0141 | 0.0497 |
| *s_Clostridium butyricum* | 7.96e-07 | 2.46e-14 | 1.17e-08 | 4.13e-15 | N.S. | N.S. | N.S. | N.S. | N.S. | N.S. |

**Supplementary Table 3**. The *p* values from Student’s *t*-test comparing short- and branched-chain fatty acids (SCFAs/BCFAs) in mmol or µmol per gram of substrates (INU, inulin; PEC, pectin; PPH, partly hydrolyzed pectin; POS, pectin oligosaccharides) at 24 h end point of CoMiniGut fermentation.

| **Organic acids** | **SUBSTRATES** | | | |
| --- | --- | --- | --- | --- |
|  | **INU** | **PEC** | **PPH** | **POS** |
| ***SCFAs (mmol/g)*** |  |  |  |  |
| Acetic acid | 0.892 | 0.897 | 0.851 | 0.421 |
| Propionic acid | 0.67 | 0.891 | 0.599 | 0.664 |
| Butyric acid | **0.031** | 0.212 | 0.169 | 0.183 |
| **Total** | **0.027** | **0.0413** | 0.15 | 0.638 |
| ***BCFAs (µmol/g)*** |  |  |  |  |
| Isobutyric acid | 0.106 | 0.129 | 0.14 | 0.69 |
| Isovaleric acid | 0.255 | 0.231 | 0.31 | 0.548 |
| 2-Methylbutyric acid | 0.205 | 0.212 | 0.346 | 0.69 |
| **Total** | 0.131 | 0.175 | 0.191 | 0.548 |
| ***Other (µmol/g)*** |  |  |  |  |
| Valeric acid | 0.318 | 0.42 | 0.841 | 0.548 |
